# Ovarian teratoma in a woodchuck (*Marmota monax*) with hepatocellular carcinoma: radiologic and pathologic features

**DOI:** 10.1186/s12917-020-02658-z

**Published:** 2020-11-23

**Authors:** Johnathan Zeng, Matthew F. Starost, Michal Mauda-Havakuk, Andrew S. Mikhail, Ari Partanen, Bradford J. Wood, John W. Karanian, William F. Pritchard

**Affiliations:** 1 Center for Interventional Oncology, Radiology and Imaging Sciences, Clinical Center, National Institutes of Health, 10 Center Drive, Room 3N320, MSC 1182, Bethesda, MD 20892 USA; 2 Division of Veterinary Resources, National Institutes of Health, Bethesda, MD 20892 USA; 3 Center for Interventional Oncology, Radiology and Imaging Sciences, Clinical Center, National Institute of Biomedical Imaging and Bioengineering and National Cancer Institute Center for Cancer Research, National Institutes of Health, Bethesda, MD 20892 USA

**Keywords:** Marmota, Teratoma, Ovarian neoplasm, Woodchuck, Pathology

## Abstract

**Background:**

Teratomas are germ cell neoplasms composed of a wide variety of tissues. In the woodchuck, only one testicular teratoma has been described in the literature. The objective of this report was to describe the radiologic and pathologic findings in a female woodchuck (*Marmota monax*) with an ovarian teratoma consisting of mature tissues originating from all three germ layers.

**Case presentation:**

A 2-year-old female woodchuck that had been infected at birth with woodchuck hepatitis virus and subsequently developed hepatocellular carcinoma was incidentally discovered to have a mobile 6.6 × 4.8 × 4.7 cm abdominal mass on computed tomography (CT) imaging. The tumor was predominantly solid and heterogenous on CT with soft tissue, fat, and areas of dense calcification. The teratoma did not enhance with intravenous contrast administration. On ultrasound, the tumor was solid with heterogeneous echogenicity, reflecting the fat content and areas of calcification. Sonolucent areas were present that may have represented cysts. There was heterogeneously increased signal on T1-weighted magnetic resonance imaging (MRI) and heterogeneous hyperintensity in T2-weighted imaging. Fat was evident within the tumor. At necropsy, the tumor was attached to the distal end of the right uterine horn. Histopathology showed mature tissue types representing all three germ layers.

**Conclusions:**

Ovarian teratoma should be considered in the differential diagnosis of ovarian or abdominal masses in woodchucks. The tumor displayed mature tissue derived from all three germ layers. CT, ultrasound, and MRI findings were presented in detail and matched the typical imaging appearance of teratomas.

## Background

There are three main types of ovarian tumors in mammals: epithelial tumors, stromal or sex cord tumors, and germ cell tumors [1]. Epithelial tumors, which include adenomas and adenocarcinomas, are the most common ovarian neoplasms. These tumors are derived from epithelium lining the ovary, including the fallopian tube and endometrium. Sex cord tumors resemble the sex cord stromal tissue of the ovary and include granulosa cell tumors, Leydig cell tumors of the ovary, and thecomas. Finally, germ cell tumors, which include teratomas and dysgerminomas, are neoplasms that mimic tissues produced by germ cells.

Teratomas are germ cell tumors with tissue derived from two or three germ layers: ectoderm, mesoderm, and endoderm. Teratomas can be immature or mature depending on the level of differentiation of each germ layer. Immature teratomas are less common and can be malignant. Mature teratomas, which are generally benign, often present with a variety of organ elements, including hair follicles, hyaline cartilage, and adipose tissue. Most often these neoplasms arise in the ovary but can also arise in the testis [1].

There have been reports of teratoma in multiple laboratory or domesticated animal species, including mice, rabbits, dogs, cats, and ferrets [2–5]. Multiple tumors have been described in the woodchuck, including lymphoma, lymphosarcoma, leiomyosarcoma, fibrosarcoma, osteosarcoma, malignant pleural mesothelioma, and meningioma [6–12]. Foley et al. reported on abnormalities in the reproductive tracts of 14 woodchucks from a series of 748 necropsies. Neoplastic lesions that were identified were uterine leiomyoma and several tumor types in the male: adenoma of the rete testis, interstitial cell tumor, seminoma, Sertoli cell tumor, lymphosarcoma, and a single case of concurrent seminoma and testicular teratoma [13, 14].

Eastern woodchucks (*Marmota monax*) infected with woodchuck hepatitis virus (WHV) have proven to be valuable pre-clinical models for the study of hepatitis B virus infection and the safety and efficacy of antiviral therapies [15, 16]. Furthermore, since hepatocellular carcinoma (HCC) naturally develops with chronic WHV infection, woodchucks are a useful model to evaluate therapies intended to treat hepatitis-induced tumors in humans [17–19]. This report describes the first case of a female woodchuck with an ovarian teratoma and presents the diagnostic imaging and pathologic findings.

## Case presentation

This study was conducted under an animal use protocol approved by the Institutional Animal Care and Use Committee in compliance with the U.S. Animal Welfare Regulations. A 2-year-old, 3.1 kg, captive-born female woodchuck (Northeastern Wildlife, Harrison, Idaho, USA) had been infected during the first week of life with WHV (cWHV7P2a inoculum, approximately 10^9^ viral particles, administered subcutaneously [20]) and subsequently developed HCC prior to acquisition for the study of HCC therapies. The animal was individually housed in the institution’s veterinary facility with 12-h light:dark cycling, provided with enrichment, and given ad libitum access to food and water.

Non-invasive diagnostic medical imaging was performed using clinical systems with the animal under general anesthesia. The animal was sedated using 5% isoflurane delivered via an induction chamber followed by a mixture of pre-anesthetics (28.6 mg/kg ketamine HCl and 5 mg/kg xylazine IM) and then maintained on 1–5% isoflurane and 100% oxygen (2 L/min) delivered via rabbit mask for the duration of the procedure. For computed tomography (CT), a 21G or 23G angiocather was inserted into a foreleg vein. At the conclusion of the planned experiments, the woodchuck was euthanized by administration of a combination of pentobarbital sodium 390 mg/mL and phenytoin sodium 50 mg/mL (Euthasol 1 mL/10 lb.; Virbac Animal Health, Fort Worth, TX, USA). The tumor was then resected and sectioned for pathology.

For CT imaging (Philips Brilliance MX8000 IDT 16-section Detector CT; Philips, Andover, MA, USA) an initial planning scan was acquired. Following non-contrast CT of the chest and abdomen, multiphase imaging of the abdomen was performed with power injection (Medrad Stellant CT Injection System, Bayer Healthcare, Leverkusen, Germany) of 3.0 mL of iopamidol i.v. (Isovue-370, Bracco Diagnostics, Monroe Township, NJ) followed by 3.0 mL 0.9% saline, all at 0.2 mL/sec. The imaging protocol was initiated once contrast appeared in the distal thoracic aorta with early arterial (4 s delay), late arterial (23 s delay), portal venous (43 s delay), and late parenchymal (63 s) phases. Scans were obtained at 120 kVp and a tube current of 225 mA with a 180 mm field of view and image reconstruction of 0.8 mm sections at 0.4 mm intervals [19, 21]. CT imaging was performed twice, 5 weeks apart. The initial CT scan was performed to define the anatomy and the extent of hepatic disease for experimental planning. The second was acquired 2 days prior to the terminal study for the purpose of designing and 3D printing a liver-specific cutting mold for the hepatic tumors [21]. Tumor measurements and image analysis were performed using OsiriX (version 11, Pixmeo, Geneva, Switzerland).

Ultrasound imaging was performed with a phased array ultrasound probe (Philips iU22, Philips Healthcare Solutions, Bothell, WA) operating at 3.75 or 5.0 MHz. Grayscale and color Doppler sonography were performed.

Magnetic resonance imaging (MRI) was performed (Achieva 3.0 T, Philips, Best, the Netherlands) with a standard 32-ch cardiac RF receive coil. MR images were acquired in the axial plane using four sequences: T1-weighted High-Resolution Isotropic Volume Examination (THRIVE), diffusion-weighted imaging (DWI), T2-weighted Turbo Spin Echo (TSE), and T2-weighted Volume Isotropic Turbo Spin Echo Acquisition (VISTA). These pulse sequences were selected to represent typical sequences used in clinical MRI liver examinations, both with and without fat suppression [21].

**Fig. 1:**
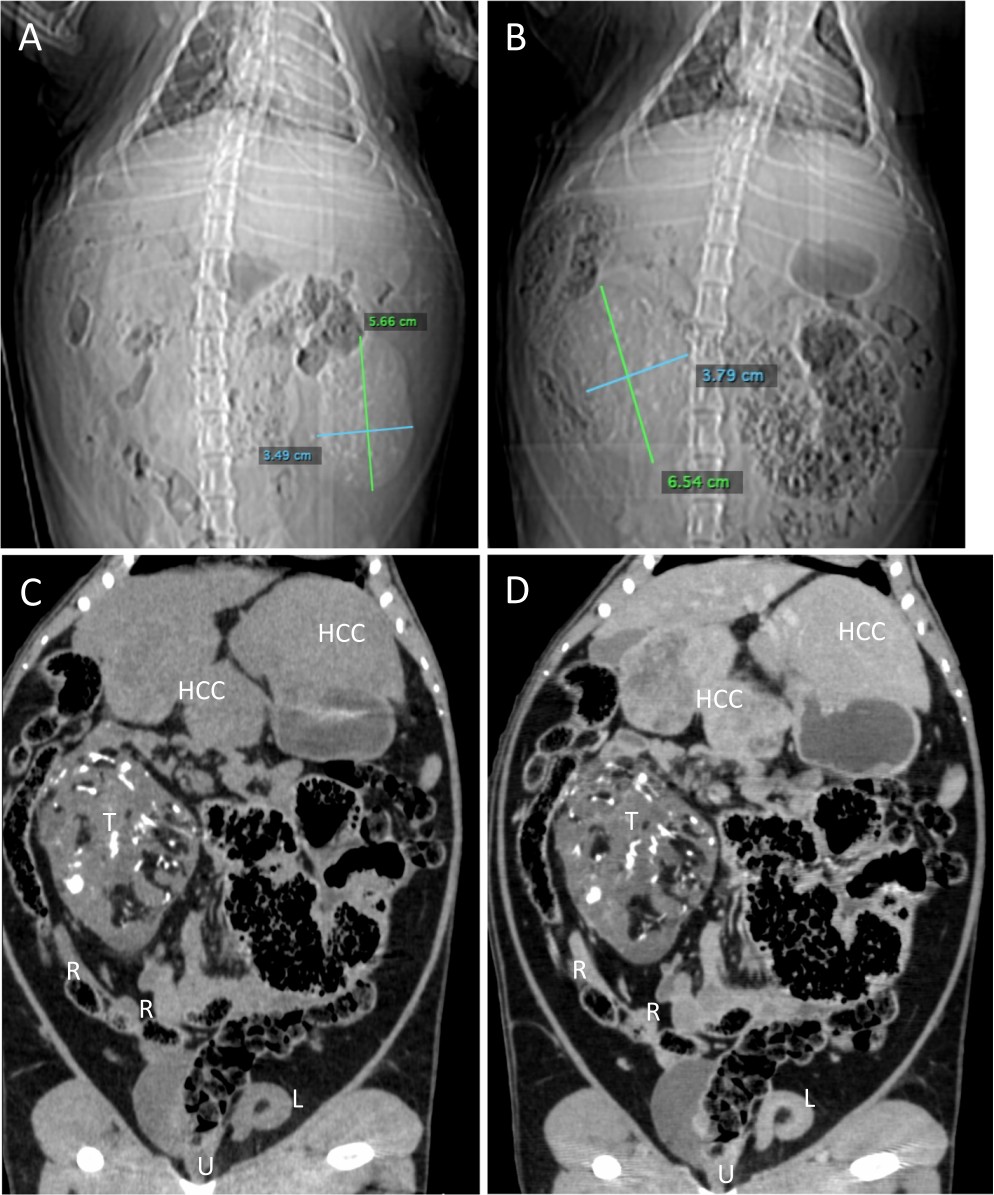
Computed Tomography. **a** Initial CT scout image and **b** CT scout image five weeks later showed a large mass with calcifications in the left abdomen on the initial scan and in the right abdomen five weeks later. Coronal reconstructions of **c** non-contrast scan and **d** 1-min delayed scan after contrast show the teratoma (T) as predominantly soft tissue density with areas of fat and dense calcification. The right uterine horn (R) is partially shown but could be traced from the body of the uterus (U) to the teratoma while the left uterine horn (L) is coiled in the caudal abdomen or pelvis. Two hepatocellular carcinomas (HCC) are shown: a 5.8 cm HCC in the left medial lobe with heterogenous enhancement and necrotic regions on this delayed image and a 3.3 cm HCC in the left lateral lobe

**Fig. 2:**
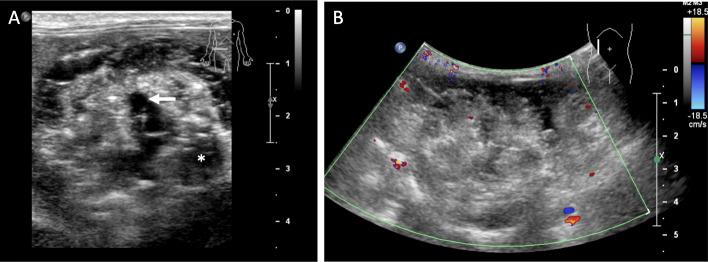
Ultrasound. **a** Grayscale ultrasound. The teratoma was solid with heterogeneous echogenicity, reflecting the fat content and areas of calcification with acoustic shadowing (asterisk) observed. Anechoic areas were present that may have represented cysts (arrow). **b**. Color Doppler ultrasound. There was little vascularity on color Doppler imaging

**Fig. 3:**
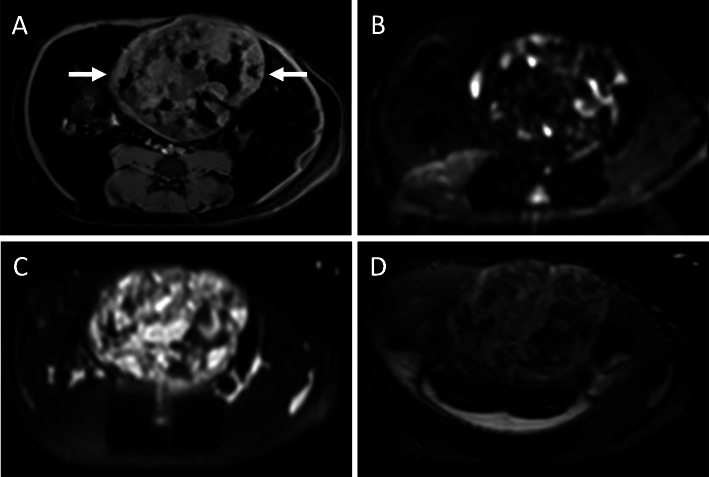
Magnetic Resonance Imaging. **a** THRIVE image of the teratoma (arrows) with heterogeneously increased signal. Signal voids in the teratoma represent fat on this fat-suppressed imaging sequence. **b** Diffusion-weighted image with focal areas of increased signal. **c** T2-weighted TSE image with heterogeneous hyperintensity. **d** T2-weighted VISTA image with heterogeneous low signal intensity

The scout images for the two CT examinations, separated by 5 weeks, showed a large calcified mass that was initially in the left abdomen measured as 5.7 × 3.5 cm (Fig. 1a) and subsequently in the right abdomen measured as 6.5 × 3.8 cm (Fig. 1b). On CT examination, the teratoma measured 6.6 × 4.8 × 4.7 cm and was well-circumscribed, predominantly solid, and heterogenous with soft tissue, fat, and areas of dense calcification (Fig. 1c, d). No fat-fluid levels were identified. Two HCCs were present, one with heterogenous enhancement and necrotic regions. Unlike the enhancement of the HCC after contrast administration, the teratoma did not enhance. The right uterine horn could be traced from the body of the uterus to the mass while the left uterine horn was coiled in the caudal abdomen and pelvis. On ultrasound, the tumor was solid with heterogeneous echogenicity, reflecting the fat content and areas of calcification with acoustic shadowing. Sonolucent areas were present that may have represented cysts. There was little vascularity on color Doppler imaging (Fig. 2). On T1-weighted THRIVE MRI, there was heterogeneously increased signal with areas of signal void representing fat. DWI showed some regions with increased signal. T2-weighted TSE indicated heterogenous hyperintensity while T2-weighted VISTA showed heterogeneous low signal intensity (Fig. 3).

**Fig. 4:**
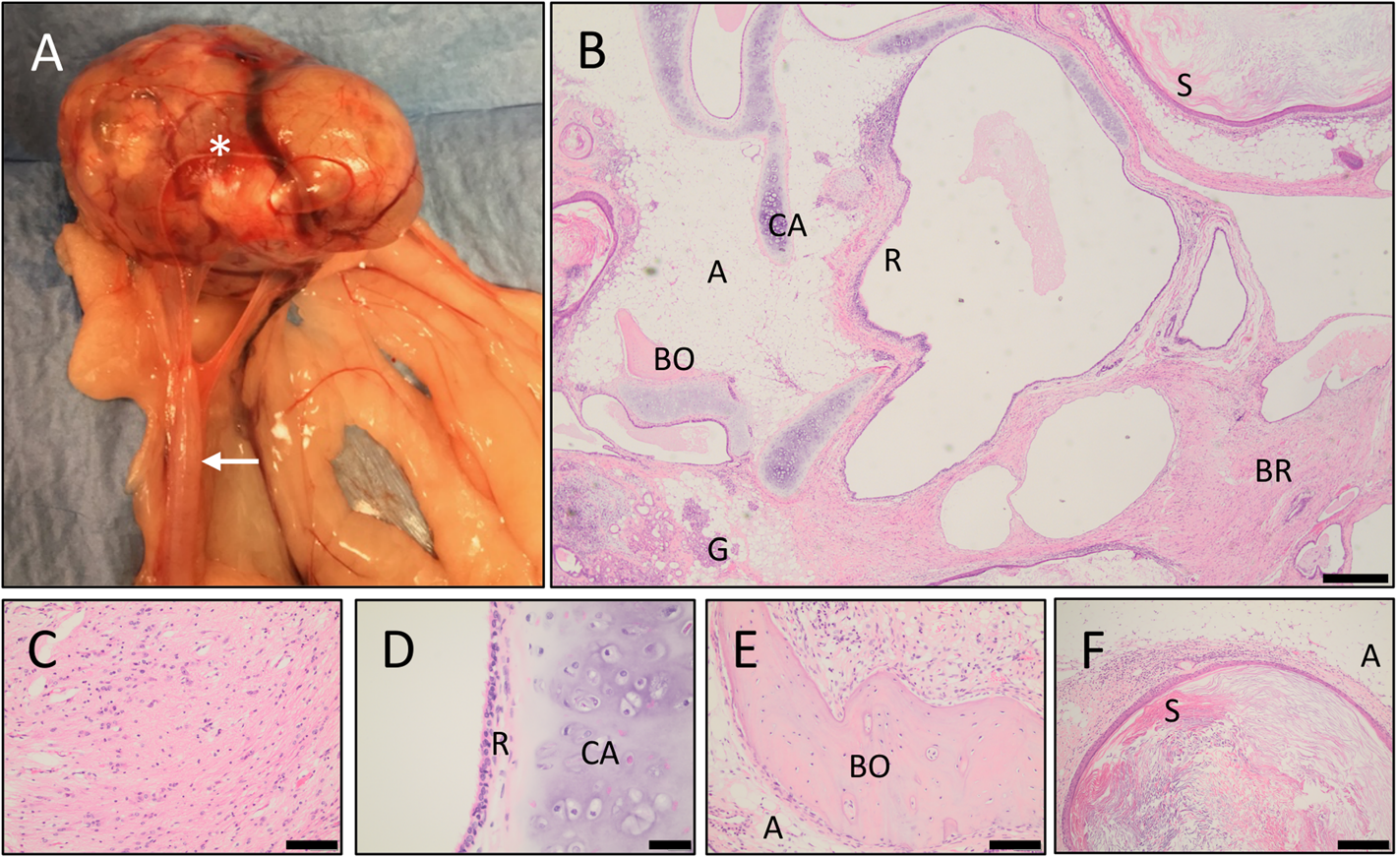
Teratoma Pathology, Gross and H&E Stains. **a** Gross specimen showing the teratoma (asterisk) attached to the uterine horn (arrow). **b** Low magnification histopathology section with multiple cell types, including brain (BR), bone (BO), cartilage (CA), white adipose tissue (A), acinar glands (G), respiratory simple columnar ciliated epithelium (R), and stratified keratinizing squamous epithelium (S). Scale bar represents 500 μm. **c** Neural tissue. Scale bar represents 100 μm. **d** Cartilage and ciliated respiratory epithelium. Scale bar represents 40 μm. **e** Mature bone matrix with adjacent adipose tissue. Scale bar represents 100 μm. **f** Keratinized stratified squamous epithelium and adipose tissue. Scale bar represents 200 μm

On gross examination following euthanasia, the teratoma was attached to the right uterine horn and was well demarcated, surrounded by a membranous translucent capsule (Fig. 4a). The HCCs were readily apparent. Histologic sections from formalin-fixed paraffin-embedded tissue samples were stained with hematoxylin and eosin. Histopathologic examination of the ovarian mass revealed tissues representing a mixture of all three germ layers: ectoderm, mesoderm, and endoderm (Table 1). Tissues from the germ layers were haphazardly arranged, although each individual tissue type could be clearly identified (Fig. 4b). Ectodermal (mesenchymal) tissue consisted of two main tissue types: neural tissue composed of neurons, glial cells, and axons; and skin with hair follicles and sebaceous glands (Fig. 4b, c). Five types of mesodermal tissue types were present: smooth muscle cells, cartilage, bone, collagen, and adipose (Fig. 4b). Mature hyaline cartilage was observed with chondrocytes interspersed within intercellular matrix adjacent to a layer of ciliated respiratory epithelium (Fig. 4d). Mature bone matrix was composed of osteocytes and contained marrow elements (Fig. 4e). Adipose tissue was seen throughout the tumor including adjacent to bone matrix and keratinized stratified squamous epithelium. Tissue of endodermal origin was present, including acinar glands and ducts. Multiple types of epithelium were present including squamous and columnar epithelium with pseudocolumnar cells lining cysts (Fig. 4b, d, f).

**Table 1:** Tissues within the teratoma grouped by germ layer of origin

Germ Layer	Tissue	Description	Figure
Ectoderm	Neural	Brain (CNS)	4B, 4C
	Skin	Hair follicles, sebaceous glands	
Ectoderm or Endoderm	Cysts	Stratified keratinizing squamous epithelium	4B, 4F
Mesoderm	Muscle	Smooth muscle cell bundles	
	Cartilage	Hyaline	4B, 4D
	Bone	Included marrow elements	4B, 4E
	Collagen	Included fibroblasts	
	Fat	White adipose tissue	4B, 4E, 4F
Endoderm	Cysts	Respiratory simple columnar and pseudocolumnar ciliated lined cysts	4B, 4D
	Cysts	Simple columnar mucus cells lining cysts	
	Cysts	Simple squamous epithelial cells	4B, 4F
	Acini	Acinar gland-like structures & duct-like structures	4B

## Discussion and conclusions

In humans, there has been variable diagnostic accuracy for teratomas among the different modalities of CT, MRI, and ultrasound. Buy et al. reported a series of cystic teratomas of the ovary and concluded that CT was the optimal diagnostic procedure [22]. They observed that fat was the most common finding in the series and argued that fat within an ovarian tumor is specific for the diagnosis. Other CT findings included Rokitansky protuberance, tooth or calcification, tufts of hair, and fat-fluid levels. In their series, 32% of the tumors were predominantly solid masses on ultrasound, as in this case. Echogenic foci with acoustic shadowing were observed. High signal intensity was observed on T1- and T2-weighted MR images in three patients. Similarly, Quillen et al. found that fat, calcifications, and low attenuation areas were the most common findings of benign cystic teratoma on CT, but could be seen uncommonly in malignant teratoma. Immature teratomas, which can demonstrate malignant characteristics, are much less common than mature teratomas [23, 24].

There is sparse reporting of clinical imaging of teratomas in small animals. The reported testicular teratoma in a woodchuck was composed of tissue derived from ectodermal and mesodermal origins partially surrounded by a seminoma. No imaging was performed [13]. While teratomas occur spontaneously in mice or rats with specific genetic mutations [25–27], imaging of teratomas has become useful in experimental studies of stem cell therapies. Riegler et al. [28] described cardiac ultrasound and MRI following transplant of human-induced pluripotent stem cells into areas of experimental myocardial infarction in rats. The utility of ultrasound was limited, but the teratomas were hyperintense on T2-weighted MRI, as in this animal. A report of imaging of a guinea pig with a palpable mass showed a 3 cm mass dorsal to the cecum with calcifications on an abdominal radiograph and irregular hyperechoic, hypoechoic, and anechoic patterns with a cystic appearance of the teratoma on ultrasound [29]. Ovarian teratomas are uncommon in cats and dogs. Mineralization within an abdominal mass on an abdominal radiograph has been reported in a teratoma in a cat [30]. Teratomas with calcification identified on abdominal radiographs or ultrasound have been reported in dogs [31–34]. Headley et al. further described the ultrasound appearance of a 22 cm teratoma in a German shepherd as a rounded, heterogenous, cystic, and echogenic mass [35]. In contrast, Gorman et al. reported an emaciated German shepherd with ascites and metastatic malignant teratoma in which the ultrasound examination also showed a hyperechoic mass and an abdominal radiograph showed mineral to bone density material within the 22 cm mass [36]. Indeed, Patnaik and Greenlee, in a series of 71 primary ovarian tumors in dogs, observed that six of seven teratomas in the series were malignant and that half of those had metastasized [37].

The appearance of the teratoma in this woodchuck was consistent with the description in humans and other small animals. The tumor in this animal was well defined with fat present which is diagnostic for teratoma in humans. The tumor was predominantly solid rather than cystic on imaging but with well-defined calcification suggesting a mature teratoma compared to coarse, ill-defined calcification that may be seen in immature teratomas in humans [24]. The diagnosis of teratoma was confirmed at pathology with mature elements representing all three germ layers. Ovarian teratoma should be considered in the differential diagnosis of ovarian or abdominal masses in woodchucks.

## Data Availability

Our findings are contained within the manuscript.

## Abbreviations

CT: Computed tomography
MRI: Magnetic resonance imaging
THRIVE: T1-weighted High-Resolution Isotropic Volume Examination
DWI: Diffusion-weighted imaging
TSE: Turbo Spin Echo
VISTA: Volume Isotropic Turbo Spin Echo Acquisition
